# Factors predicting recurrence after left‑sided pancreatectomy for pancreatic ductal adenocarcinoma

**DOI:** 10.1186/s12957-023-03080-z

**Published:** 2023-06-22

**Authors:** Tao Xia, Peng Xu, Yiping Mou, Xizhou Zhang, Shihao Song, Yucheng Zhou, Chao Lu, Qicong Zhu, Yunyun Xu, Weiwei Jin, Yuanyu Wang

**Affiliations:** 1 Department of General Surgery, Cancer Center, Division of Gastrointestinal and Pancreatic Surgery, Zhejiang Provincial People’s Hospital, Affiliated People’s Hospital, Hangzhou Medical College, Hangzhou, China; 2 Department of Surgery, Qingdao University, Qingdao, China; 3 Department of Surgery, Wenzhou Medical University, Wenzhou, China

**Keywords:** Left‑sided pancreatectomy, Pancreatic ductal adenocarcinoma, Recurrence site, Recurrence-free survival, Predictors for recurrence

## Abstract

**Background:**

Recurrence after resection is the main factor for poor survival. The relationship between clinicopathological factors and recurrence after curative distal pancreatectomy for PDAC has rarely been reported separately.

**Methods:**

Patients with PDAC after left‑sided pancreatectomy between May 2015 and August 2021 were retrospectively identified.

**Results:**

One hundred forty-one patients were included. Recurrence was observed in 97 patients (68.8%), while 44 (31.2%) patients had no recurrence. The median RFS was 8.8 months. The median OS was 24.9 months. Local recurrence was the predominant first detected recurrence site (*n* = 36, 37.1%), closely followed by liver recurrence (*n* = 35, 36.1%). Multiple recurrences occurred in 16 (16.5%) patients, peritoneal recurrence in 6 (6.2%) patients, and lung recurrence in 4 (4.1%) patients. High CA19-9 value after surgery, poor differentiation grade, and positive lymph nodes were found to be independently associated with recurrence. The patients receiving adjuvant chemotherapy had a decreased likelihood of recurrence. In the high CA19-9 value cohort, the median PFS and OS of the patients with or without chemotherapy were 8.0 VS. 5.7 months and 15.6 VS. 13.8 months, respectively. In the normal CA19-9 value cohort, there was no significant difference in PFS with or without chemotherapy (11.7 VS. 10.0 months, *P* = 0.147). However, OS was significantly longer in the patients with chemotherapy (26.4 VS. 13.8 months, *P* = 0.019).

**Conclusions:**

Tumor biologic characteristics, such as T stage, tumor differentiation and positive lymph nodes, affecting CA19-9 value after surgery are associated with patterns and timing of recurrence. Adjuvant chemotherapy significantly reduced recurrence and improved survival. Chemotherapy is strongly recommended in patients with high CA199 after surgery.

## Introduction

Pancreatic ductal adenocarcinoma (PDAC) is an aggressive malignancy with a low survival rate despite improved multimodality treatment. Radical resection and systemic therapy are the only chance to provide long-term survival [1, 2]; however, recurrence after resection exceeds 70–80% and is the main factor for poor survival [3, 4]. Understanding the patterns and timing of disease recurrence can help guide improvements in therapy.

Several prior studies have reported on the patterns of recurrence following resection for PDAC and suggested that different sites of recurrence carry different survival rates [5–7]. Groot et al. reported that local recurrence occurred in 23.7%, liver-only recurrence in 25.2%, local + distant in 18.5%, and multiple in 4.7%. Patients with multisite and liver recurrence had worse survival than those with local or pulmonary recurrence. Identifying the risk factors that can predict recurrence sites and timing of recurrence have potential clinical applications for prognostic stratification to determine whether to perform more aggressive therapy [8–10]. Increasing evidence has shown that PDAC at the head and tail displays different clinical presentations, treatment efficiencies and prognoses [11, 12]. A retrospective analysis of 616 patients with PDAC who underwent surgical resection reported that patients undergoing distal pancreatectomy for left-sided lesions had larger tumors (4.7 vs. 3.1 cm, *P* < 0.0001) but fewer positive nodes (59% vs. 73%, *P* = 0.03) and fewer poorly differentiated tumors (29% vs. 36%, *P* < 0.001) than those undergoing pancreaticoduodenectomy for right-sided lesions [13]. Previous reports including patients who have had pancreatectomy for PDAC have not distinguished between lesions in the pancreatic head and tail. The relationship between clinicopathological factors and recurrence after curative distal pancreatectomy for PDAC has rarely been reported separately.

Therefore, this study aimed to establish the patterns of recurrence and timing of disease recurrence survival following left‑sided pancreatectomy for PDAC. Furthermore, perioperative risk factors for correlation with recurrence sites and time were identified.

## Materials and methods

### Patients

Patients with left‑sided pancreatic ductal adenocarcinoma who underwent surgical resection at Zhejiang Provincial Peoples’ Hospital between May 2015 and August 2021 were retrospectively identified. All acquisition methods were approved by the Institutional Review Board. Patients with left-sided PDAC confirmed by postoperative pathology were included. Patients with 90-day postoperative mortality, with vascular resection and with less than 12 months of follow-up in which neither recurrence nor death occurred were excluded. Follow-up was stopped in September 2022.

### Characteristics and definitions

Demographic, clinicopathologic and treatment data were collected. Resectability and staging were evaluated using pancreatic computed tomography angiography (CTA) and were typically discussed in a multidisciplinary team (MDT). Patients with either locally advanced or borderline resectable disease were preferentially referred for neoadjuvant therapy. Chemotherapy was recommended as routine therapy for patients with a performance status of 0–1 Eastern Cooperative Oncology Group (ECOG). Chemotherapy regimens and duration were left to the discretion of the oncologist in the MDT. After neoadjuvant chemotherapy with at least stable disease (SD), the decision for operative exploration was at the pancreatic surgeon’s discretion in the MDT. Pathological data were classified using the 8th AJCC/UICC TNM staging system [14]. R1 margin status was defined as ≤ 1 mm from the edge of the specimen which was reported by two pathologists. A high carbohydrate antigen (CA) 19–9 value was > 37 U/L according to our hospital laboratory threshold, and a normal CA19-9 value was ≤ 37 U/L.

After completion of all therapy, patient follow-up occurred by computed tomography (CT) every 3 months within the first 2 years and every 6 months if no recurrence was detected. Recurrence-free survival (RFS) was defined as the time between the date of operation to the date of recurrence and was the primary outcome measure. Overall survival (OS) was defined as the time between surgery and either the date of death or last follow-up. Recurrence was defined based on the imaging findings. In most instances, a tissue biopsy was performed to confirm the lesion if the biopsy was easy to perform with B ultrasound. Local recurrence was defined as recurrence in the stump of the pancreas or in the surgical bed, such as soft tissue along the peripancreatic vasculature (the celiac or superior mesenteric artery). Distant recurrence was defined as metastasis to the liver, peritoneum and lung. Multiple recurrence was defined as local plus distant sites and multiple distant sites.

### Statistical analysis

All statistical analyses were performed using SPSS v.22.0 and GraphPad Prism 8 software. Continuous variables were compared using Student’s t test or the Wilcoxon rank test for parametric or nonparametric distributions, respectively. Categorical variables were compared using the chi-square or Fisher’s exact test. Binary logistic regression was performed to evaluate factors for recurrence. OS and RFS were assessed using the Kaplan–Meier estimate method with corresponding 95% confidence intervals (95% CI), and comparisons were conducted using the log-rank test. Only variables with p values less than 0.15 in the univariate analysis were included in the multivariate regression analysis to identify independent prognostic factors. P values less than 0.05 were considered statistically significant.

## Results

### Patient characteristics

A total of 141 patients were included and 2 patients was excluded for postoperative hemorrhage death within 90-day. Because two patients had a pathological complete response, some data only had 139 values. The median age was 65 (IQR 38–88) years, and the mean BMI was 22.15 ± 2.98 kg/m^2^. The mean tumor size was 3.5 (0.5–10) cm. The demographic, clinicopathological and treatment characteristics for the patients with and without recurrence are presented separately in Table 1.

**Table 1:** Demographics, clinicopathological, and treatment characteristics of patients with or without recurrence

Variables	All patients (*N* = 141)	Recurrence (*N* = 97)	No recurrence (*N* = 44)
Age(years)			
≤ 74	116(82.3%)	79(81.4%)	37(84.1%)
≥ 75	25(17.7%)	18(18.6%)	7(15.9%)
Sex (%)			
Male	80(56.7%)	59(60.8%)	21(47.7%)
Female	61(43.3%)	38(39.2%)	23(52.3%)
BMI (kg/m^2^)			
≤ 18.4	10(7.1%)	6(6.2%)	4(9.1%)
≥ 18.5	131(92.9%)	91(93.8%)	40(90.9%)
Surgical Procedure			
RAMPS	67(47.5%)	43(44.3%)	24(54.5%)
DP	74(52.5%)	54(55.7%)	20(45.5%)
Preoperative CA19-9 (U/L)			
≤ 37	32(22.7%)	19(19.6%)	13(29.5%)
> 37	109(77.3%)	78(80.4%)	31(70.5%)
Postoperative CA19-9 (U/L)			
≤ 37	81(57.4%)	48(49.5%)	33(75%)
> 37	60(42.6%)	49(50.5%)	11(25%)
T stage			
T_1-2_	93(66.0%)	63(64.9%)	30(68.2%)
T_3-4_	48(34.0%)	34(35.1%)	14(31.8%)
N stage			
N_0_	91(64.5%)	55(56.7%)	36(81.8%)
N_1-2_	50(35.5%)	42(43.3%)	8(18.2%)
Tumor differentiation			
Well /moderate	71(51.1%)	46(47.4%)	25(59.5%)
Poor	68(48.9%)	51(52.6%)	17(40.5%)
R-status			
R1	6(4.3%)	4(4.1%)	2(4.8%)
R0	133(95.7%)	93(95.9%)	40(95.2%)
Perineural invasion			
Yes	110(79.1%)	76(78.4%)	34(81.0%)
No	29(20.9%)	21(21.6%)	8(19.0%)
Lymphovasular invasion			
Yes	51(36.7%)	37(38.1%)	14(33.3%)
No	88(63.3%)	60(61.9%)	28(66.7%)
Neoadjuvant chemotherapy			
Yes	14(9.9%)	12(12.4%)	2(4.5%)
No	127(90.1%)	85(87.6%)	42(95.5%)
Adjuvant chemotherapy			
Yes	82(58.2%)	51(52.6%)	31(70.5%)
No	59(41.8%)	46(47.4%)	13(29.5%)

Most patients had resectable tumors at diagnosis (127, 90.1%). Fourteen (9.9%) patients received neoadjuvant chemotherapy (NACT), and the regimens included FOLFIRINOX (4 of 14, 28.6%) and gemcitabine-nab-paclitaxel (10 of 14, 71.4%). The median duration of NACT was 4.0 (IQR 2.1–6.0) months. Two patients had a pathological complete response using FOLFIRINOX. Adjuvant therapy was administered in 82 (58.2%) patients. The most common regimens were gemcitabine-nab-paclitaxel (52 of 82, 63.4%), gemcitabine-5-flurouracil (16 of 82, 19.5%), FOLFIRINOX (11 of 82, 13.4%), and single-agent gemcitabine (3 of 82, 3.7%).

### Survival and factors associated with recurrence

The median follow-up was 50.4 (95% CI 36.45–64.35) months. Recurrence was observed in 97 patients (68.8%), while 44 (31.2%) patients had no recurrence. The median RFS was 8.8 (95% CI 7.51–10.09) months. The median OS was 24.9 (95% CI 17.44–32.30) months. The median OS was 16.8 (95% CI 13.92–19.68) months in the recurrence cohort, and the median OS was not reached in the recurrence-free cohort.

Several prognostic clinicopathological factors were found to be independently associated with an increased likelihood of recurrence: high CA19-9 value after surgery (HR 2.22, 95% CI 1.49–3.32, *P* = 0.000), poor differentiation grade (HR 1.59, 95% CI 1.07–2.38, *P* = 0.023), and positive lymph nodes (HR 1.80, 95% CI 1.20–2.70, *P* = 0.005). The patients receiving adjuvant chemotherapy (HR 0.59, 95% CI 0.40–0.88, *P* = 0.010) had a decreased likelihood of recurrence. A subsequent multivariate analysis was further performed. High CA19-9 values after surgery (HR 2.16, 95% CI 1.44–3.14, *P* = 0.000) and positive lymph nodes (HR 1.69, 95% CI 1.12–2.53, *P* = 0.012) were independent poor predictive factors for recurrence. All data are shown in Table 2.

**Table 2:** Univariate and multivariate of predictive factors for recurrence

Variables	Univariate			Multivariate		
	HR	95% CI	*P* value	HR	95% CI	*P* value
Sex (Female / Male)	0.73	0.48–1.09	0.125	0.74	0.49–1.11	0.143
Age (≤ 74/ ≥ 75 years)	0.87	0.52–1.45	0.579			
BMI (≤ 18.4/ ≥ 18.5 kg/m^2^)	0.78	0.34–1.78	0.555			
Preoperative CA19-9 (> 37/ ≤ 37U/L)	1.57	0.95–2.60	0.079	1.09	0.55–1.78	0.959
Postoperative CA19-9 (> 37/ ≤ 37U/L)	2.22	1.49–3.32	0.000	2.16	1.44–3.14	0.000
LDPS / LRAMPS	1.23	0.82–1.84	0.309			
T(T_1-2_/T_3-4_)	0.95	0.62–1.43	0.792			
Positive lymph nodes (Yes / No)	1.80	1.20–2.70	0.005	1.69	1.12–2.53	0.012
R- status (R1 /R0)	1.34	0.49–3.64	0.671			
Tumor differentiation (Poor/Well-moderate)	1.59	1.07–2.38	0.023	1.21	0.79–1.86	0.379
Perineural invasion (Yes/No)	1.11	0.68–1.80	0.679			
Lymphovasular invasion (Yes/No)	1.11	0.73–1.69	0.636			
Adjuvant chemotherapy (Yes/No)	0.60	0.40–0.88	0.010	0.74	0.49–1.11	0.143

**Fig. 1:**
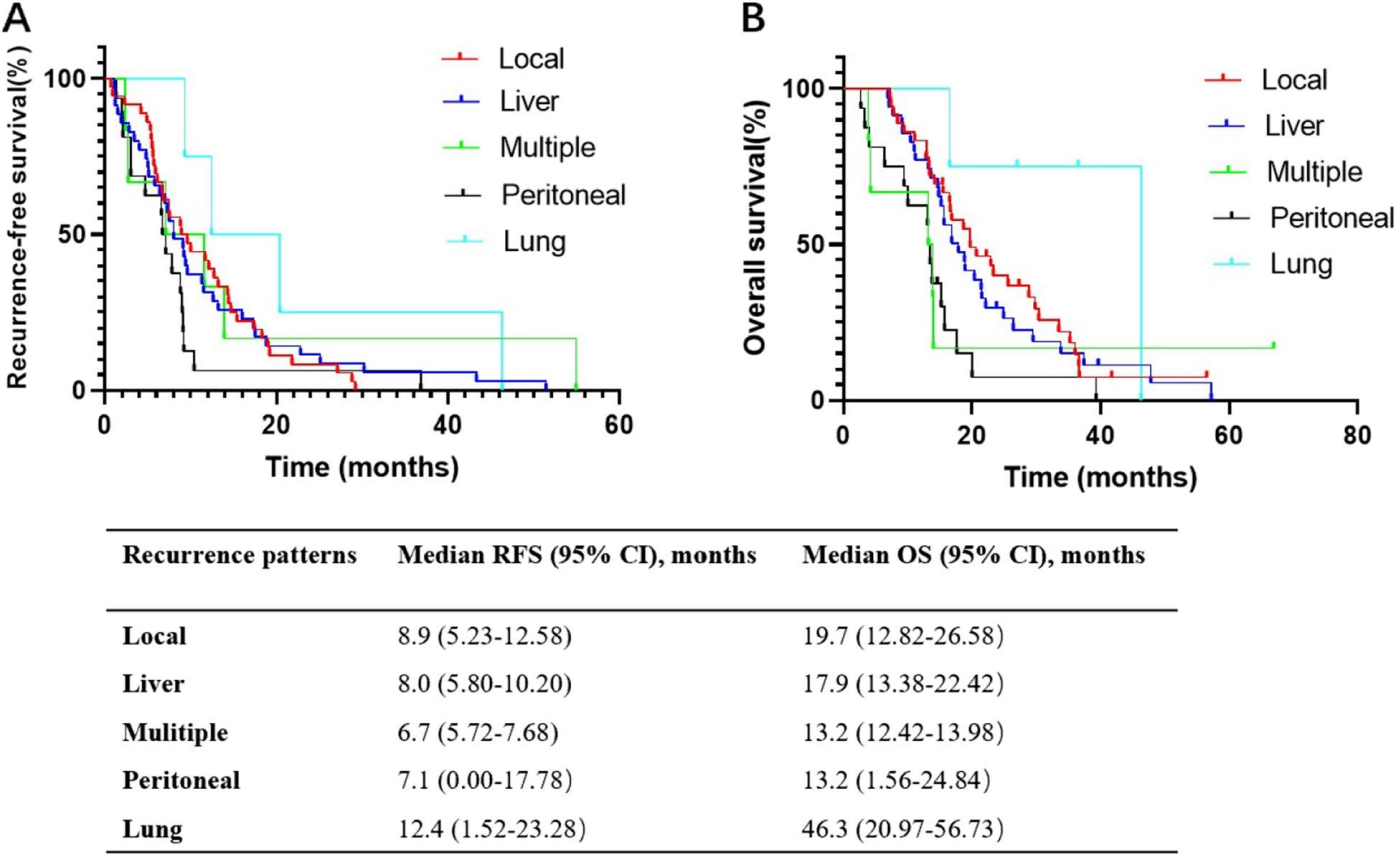
**A** Recurrence-free survival (RFS) of different recurrence sites. **B** Overall survival (OS) curves of different recurrence sites

Local recurrence was the predominant first detected recurrence site (*n* = 36, 37.1%), which was closely followed by liver recurrence (*n* = 35, 36.1%). Multiple recurrences occurred in 16 (16.5%) patients, peritoneal recurrence in 6 (6.2%) patients, and lung recurrence in 4 (4.1%) patients. A pairwise comparison of the median RFS and OS between the specific site recurrence patterns is shown in Fig. 1. Multiple recurrence had the shortest RFS (6.7 months, 95% CI 5.72–7.68), and pulmonary recurrence had the longest RFS (12.4 months, 95% CI 1.52–23.28). The median OS for the patients with local, liver recurrence, multiple recurrence and distant recurrence, and peritoneal recurrence exceeded 12 months and did not differ significantly from each other. The patients with pulmonary recurrence had the longest OS (46.3 months, 95% CI 20.97–56.73).

Due to the sample size, analyses for identifying potential factors predicting patterns of recurrence were performed in local-only and liver-only recurrence, as shown in Tables 3 and 4, respectively. A high CA19-9 value after surgery was the only independent poor predictive factor for local recurrence (HR 2.24, 95% CI 1.12–4.47, *P* = 0.023). High CA19-9 values after surgery (HR 2.31, 95% CI 1.04–5.10, *P* = 0.039) and poor differentiation grade (HR 2.36, 95% CI 1.05–5.34, *P* = 0.038) were statistically associated with liver recurrence. Adjuvant chemotherapy significantly reduced liver recurrence (HR 0.42, 95% CI 0.19–0.91, *P* = 0.028).

**Table 3:** Univariate and multivariate of predictive factors for local recurrence

Variables	Univariate			Multivariate		
	HR	95% CI	*P* value	HR	95% CI	*P* value
Sex (Female / Male)	0.62	0.31–1.22	0.165			
Age (≤ 74/ ≥ 75 years)	1.36	0.52–3.54	0.526			
BMI (≤ 18.4/ ≥ 18.5 kg/m^2^)	1.64	0.22–12.39	0.632			
Preoperative CA19-9 (> 37/ ≤ 37U/L)	1.41	0.58–3.44	0.443			
Postoperative CA19-9 (> 37/ ≤ 37U/L)	2.24	1.12–4.47	0.023	2.24	1.12–4.47	0.023
LDPS / LRAMPS	1.63	0.82–3.25	0.164			
T(T_1-2_/T_3-4_)	0.86	0.43–1.74	0.679			
Positive lymph nodes (Yes / No)	1.14	0.53–1.80	0.933			
R- status (R1 /R0)	2.47	0.73–8.40	0.147	1.33	0.36–4.95	0.671
Tumor differentiation (Poor/Well-moderate)	1.51	0.75–3.04	0.247			
Perineural invasion (Yes/No)	1.47	0.56–3.38	0.432			
Lymphovasular invasion (Yes/No)	0.65	0.33–1.29	0.220			
Adjuvant chemotherapy (Yes/No)	0.59	0.30–1.17	0.130	0.67	0.33–1.34	0.253

**Table 4:** Univariate and multivariate of predictive factors for liver recurrence

Variables	Univariate			Multivariate		
	HR	95% CI	*P* value	HR	95% CI	*P* value
Sex (Female / Male)	0.96	0.46–2.20	0.919			
Age (≤ 74/ ≥ 75 years)	0.84	0.34–2.06	0.694			
BMI (≤ 18.4/ ≥ 18.5 kg/m^2^)	0.58	0.14–2.45	0.459			
Preoperative CA19-9 (> 37/ ≤ 37U/L)	1.45	0.50–4.19	0.496			
Postoperative CA19-9 (> 37/ ≤ 37U/L)	2.33	1.11–4.89	0.025	2.31	1.04–5.10	0.039
LDPS / LRAMPS	0.40	0.19–0.87	0.220			
T(T_1-2_/T_3-4_)	0.86	0.40–1.84	0.692			
Positive lymph nodes (Yes / No)	1.34	0.61–2.94	0.473			
R- status (R1 /R0)	1.91	0.13–1.89	0.239			
Tumor differentiation (Poor/Well-moderate)	2.95	1.33–6.54	0.008	2.36	1.05–5.34	0.038
Perineural invasion (Yes/No)	2.15	0.91–5.07	0.179			
Lymphovasular invasion (Yes/No)	1.01	0.47–2.40	0.877			
Adjuvant chemotherapy (Yes/No)	0.47	0.23–0.99	0.048	0.42	0.19–0.91	0.028

### The significance of the CA19-9 value after surgery

**Fig. 2:**
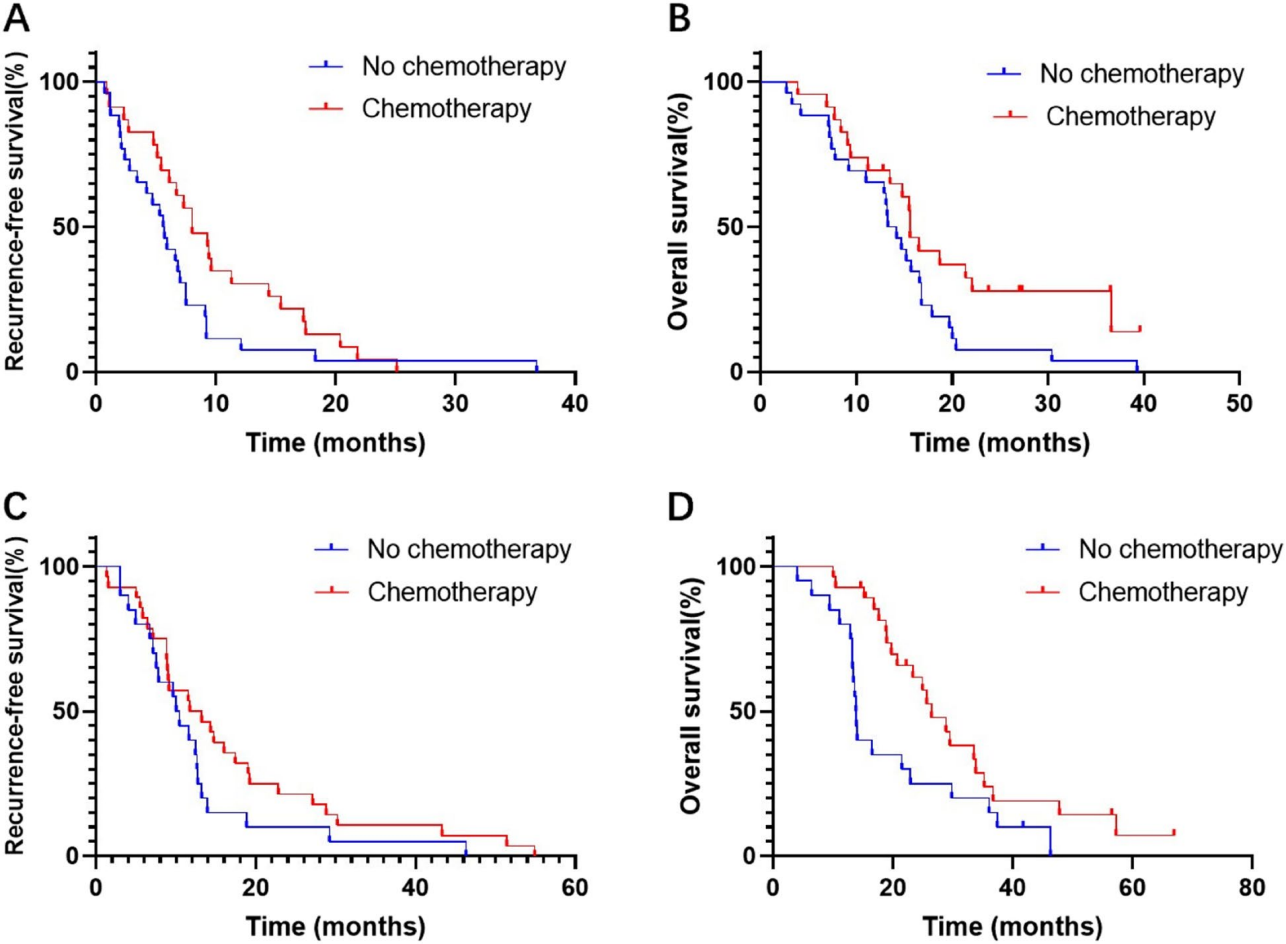
**A** Recurrence-free survival (RFS) of patients with or without chemotherapy in high CA19-9 value after surgery cohort. **B** Overall survival (OS) curves of patients with or without chemotherapy in high CA19-9 value after surgery cohort. **C** Recurrence-free survival (RFS) of patients with or without chemotherapy in normal CA19-9 value after surgery cohort. **D** Overall survival (OS) curves of patients with or without chemotherapy in normal CA19-9 value after surgery cohort

A high CA19-9 value after surgery was found to be independently associated with an increased likelihood of recurrence. CA19-9 value after surgery in patients with recurrence was further analyzed as shown in Table 5. T stage and tumor differentiation were statistically correlated with the CA19-9 value after surgery. The median RFS and OS of the patients with high CA19-9 value after surgery were significantly shorter than those of the patients with normal CA19-9 value. In the high CA19-9 value cohort, the median PFS (*P* = 0.041) and OS (*P* = 0.048) of the patients with or without chemotherapy were 8.0 (95% CI 4.87–11.13) vs. 5.7 (95% CI 4.10–7.10) months and 15.6 (95% CI 14.11–17.10) vs. 13.8 (95% CI 11.30–15.30) months, respectively (Fig. 2 A, B). In the normal CA19-9 value cohort, there was no significant difference in PFS in the patients with or without chemotherapy (11.7 (95% CI 4.93–18.41) vs. 10.0 (95% CI 8.25–11.75 months, *P* = 0.147). However, OS was significantly longer in the patients with chemotherapy (26.4 (95% CI 20.92–31.95) vs. 13.8 (95% CI 13.14–14.46) months, *P* = 0.019) (Fig. 2 C, D).

**Table 5:** Clinicopathological and treatment characteristics associated with CA19-9 value after surgery

Variables	High CA19-9(*N* = 49)	Normal CA19-9 (*N* = 48)	*P* value
Age(years)			0.319
≤ 74	38(77.6%)	41(85.4%)	
≥ 75	11(22.4%)	7(14.6%)	
Sex (%)			0.184
Male	33(67.3%)	26(54.2%)	
Female	16(32.7%)	22(45.8%)	
BMI (kg/m^2^)			0.414
≤ 18.4	4(8.2%)	2(4.2%)	
≥ 18.5	45(91.8%)	46(95.8%)	
Surgical procedure			
RAMPS	25(51%)	18(37.5%)	0.180
DP	24(29%)	30(62.5%)	
T stage			
T_1-2_	27(55.1%)	36(75%)	0.040
T_3-4_	22(44.9%)	12(25%)	
N stage			
N_0_	25(51%)	30(62.5%)	0.254
N_1-2_	24(29%)	18(37.5%)	
Tumor differentiation			
Well /moderate	18(36.7%)	29(60.4%)	0.020
Poor	31(63.3%)	19(39.6%)	
R-status			0.316
R1	3 (6.1%)	1(2.1%)	
R0	46(93.9%)	46(97.9%)	
Perineural invasion			0.075
Yes	42(85.7%)	34(70.8%)	
No	7(14.3%)	14(29.2%)	
Lymphovasular invasion			0.334
Yes	21(42.9%)	16(33.3%)	
No	28(57.1%)	32(66.7%)	
PFS (months)	6.8 (95%CI 5.04–8.56)	12.6 (95%CI 10.52–14.68)	0.002
OS (months)	15.2 (95%CI 13.22–17.25)	22.9 (95%CI 16.41–29.38)	0.003

## Discussion

Several prior clinical studies have reported recurrence following pancreatectomy for PDAC regardless of adjuvant chemotherapy. The recurrence rate differed between the included clinical studies and ranged from 54.7% to 91.1% [4, 6, 15]. Prognostic factors for PDAC after surgical resection are established, including resection margin [16], tumor markers [17, 18], tumor differentiation [9], and chemotherapy [9, 19]. However, recurrence after curative distal pancreatectomy for PDAC has rarely been reported separately. This report presents a single large institutional study of patterns and timing of recurrence following left‑sided pancreatectomy for PDAC in the same multidisciplinary team (MDT). In this cohort, 97 (68.8%) patients had recurrence after resection, and the median RFS was 8.8 months. The recurrence rate and median RFS are similar to previous larger sample size studies of pancreatectomy for PDAC [8], despite different biological behaviors between the head and tail.

In this current cohort, the most common site was local recurrence (37.1%), followed by liver recurrence (36.1%), multiple recurrence (16.5%), peritoneal recurrence (6.2%), and pulmonary recurrence (4.1%). The RFS for local recurrence (median 9.3 months) and liver recurrence (8 months) are comparable, which was similar to a report by Sperti et al., which presented RFS for local recurrence (median 9.5 months) and liver recurrence (9.0 months) [20]. A recurrence analysis of the ESPAC-4 randomized adjuvant chemotherapy trial showed that local recurrence occurred at a median of 11.63 months, which was significantly different from distant recurrence with a median of 9.49 months. Furthermore, the median overall survival of patients with distant-only recurrence (23.03 months) or local recurrence with distant recurrence (23.82 months) was not significantly different from that of patients with only local recurrence (24.83 months) [21]. In our study, the OS of liver recurrence (17.9 months) was not significantly different from that of local recurrence (19.7 months). In addition, patients with multiple recurrences had the shortest RFS (6.9 months) and OS (13.4 months). Patients with lung recurrence had the longest RFS (12.4 months) and OS (46.3 months). The RFS and OS of patients with peritoneal recurrence were 9.35 months and 13.2 months, respectively. Recurrence location and timing showed great variation due to heterogeneous biological behavior and can reflect tumor aggressiveness. Multiple recurrences occur early and have a poor prognosis. Local recurrence can be recognized early due to the symptoms but with an uninspiring survival, which may be because symptomatic recurrence is associated with aggressive tumor biology [4]. Peritoneal recurrence, which is sensitive to expensive positron emission tomography-computed tomography (PET-CT), cannot be detected earlier by cross-sectional computed tomography imaging [22]. Several reports have demonstrated that lung recurrence has a better PFS and prognosis due to slower growth and less aggressiveness [8]. Daishi et al. reported that the lung metastasis rate was 7.5%; furthermore, the median RFS and OS were 18.2 months and 86.4 months, respectively. In addition, they found that lung metastasis had a high proportion of PDAC of the body and tail and a high frequency of arterial invasion because of spreading through the portosystemic shunt to extra-abdominal organs [23].

A further aim of this study was to establish clinicopathological features correlated with the timing and patterns of recurrence. High CA19-9 value after surgery, poor differentiation grade and positive lymph nodes were found to be independently associated with an increased likelihood of recurrence in general. A high CA19-9 value after surgery was the only independent poor predictive factor for local recurrence. Furthermore, a high CA19-9 value after surgery and poor differentiation grade were statistically associated with liver recurrence. Kolbeinsson et al. suggest that patients with poorly differentiated tumors are over 4 times more likely to experience recurrence in the liver [9]. Groot et al. found that poor tumor differentiation was associated with the development of multiple recurrences and hepatic recurrence [8].

In our study, a high CA19-9 value after surgery was found to be an important factor associated with recurrence both in general and at the site, among many statistically significant factors. The median RFS and OS of patients with high CA19-9 values after surgery were significantly shorter than those of patients with normal CA19-9 values. Currently, CA19-9 is the most widely used serum biomarker for the diagnosis and prognosis of PDAC. Many studies have investigated the role of CA19-9 in the prediction of postresection outcomes [22]. A meta-analysis demonstrated that elevated CA19-9 (> 305 KU/L) levels were independently associated with poor OS (HR: 1.72 (1.31–2.26)) and early recurrence (HR: 1.74 (1.06–2.86)) in PDAC patients [24]. Maggino et al. reported that preoperative tumor size < 20 mm and normal post-treatment CA19-9 were associated with longer RFS following post-neoadjuvant pancreatectomy in initially resectable and borderline resectable PDAC [17]. On further analysis of the current cohort, T stage and tumor differentiation were statistically correlated with the CA19-9 value after surgery. These tumor-associated biological characteristics, which affect the CA19-9 value after surgery, are also risk factors for recurrence. Because inherent factors cannot be altered, other methods, such as treatment methods, have been explored to improve survival.

A prior study reported that chemotherapy (HR 0.75, 95% CI 0.57–0.97, *P* = 0.027) and chemoradiotherapy (HR 0.73, 95% CI 0.61–0.89, *P* = 0.001) significantly reduce the likelihood of recurrence in general [8]. A recent study showed similar results, identifying that receipt of 6 or more cycles of chemotherapy as part of first-line therapy correlated with improved survival [9]. A propensity score-matched SEER database analysis revealed chemotherapy as a protective prognostic factor for survival [25]. The current study showed that adjuvant chemotherapy significantly reduced recurrence and improved survival. Liver recurrence was reduced after adjuvant chemotherapy, which may be explained by the fact that PDAC is considered a systemic disease, and additional chemotherapy is often recommended to prolong OS after resection. Patients with high CA199 values after surgery should be considered the most suitable candidates for chemotherapy to prolong PFS and OS. In addition, chemotherapy improved the survival of patients with normal CA199 values after surgery.

Several limitations in this study need to be addressed. First, patients receive multimodal therapy during a long follow-up period. Therefore, the chemotherapy regimens and cycles are diverse and can be lacking. Second, treatments after recurrence are lacking, which affects overall survival, so an analysis of clinicopathological features correlated with OS was not performed.

## Conclusions

This study provides the timing and pattern of recurrence after distal pancreatectomy for left-sided PDAC. Furthermore, clinicopathological features were identified to predict RFS of general and different sites. Tumor biologic characteristics such as T stage, tumor differentiation and positive lymph nodes affecting CA19-9 value after surgery are associated with patterns and timing of recurrence. Adjuvant chemotherapy significantly reduced recurrence and improved survival. Furthermore, chemotherapy is strongly recommended in patients with high CA199 after surgery. These findings are highly suggestive of biological heterogeneity among individuals with PDAC.

## Data Availability

The data presented in this study are available on request from the corresponding author.

## References

[1] Bengtsson A, Andersson R, Ansari D (2020). The actual 5-year survivors of pancreatic ductal adenocarcinoma based on real-world data [J]. Sci Rep.

[2] Sohal DPS, Duong M, Ahmad SA, Gandhi NS, Beg MS, Wang-Gillam A (2021). Efficacy of Perioperative Chemotherapy for Resectable Pancreatic Adenocarcinoma: A Phase 2 Randomized Clinical Trial [J]. JAMA Oncol.

[3] Malleo G, Maggino L, Ferrone CR, Marchegiani G, Warshaw AL, Lillemoe KD (2020). Reappraising the Concept of Conditional Survival After Pancreatectomy for Ductal Adenocarcinoma: A Bi-institutional Analysis [J]. Ann Surg.

[4] Daamen LA, Groot VP, Besselink MG, Bosscha K, Busch OR, Cirkel GA (2022). Detection, Treatment, and Survival of Pancreatic Cancer Recurrence in the Netherlands: A Nationwide Analysis [J]. Ann Surg.

[5] Van Den Broeck A, Sergeant G, Ectors N, Van Steenbergen W, Aerts R, Topal B. Patterns of recurrence after curative resection of pancreatic ductal adenocarcinoma [J]. European journal of surgical oncology : the journal of the European Society of Surgical Oncology and the British Association of Surgical Oncology. 2009;35(6):600–4. 10.1016/j.ejso.2008.12.006. 10.1016/j.ejso.2008.12.006 19131205

[6] Neoptolemos JP, Stocken DD, Friess H, Bassi C, Dunn JA, Hickey H (2004). A randomized trial of chemoradiotherapy and chemotherapy after resection of pancreatic cancer [J]. N Engl J Med.

[7] Schnelldorfer T, Ware AL, Sarr MG, Smyrk TC, Zhang L, Qin R (2008). Long-term survival after pancreatoduodenectomy for pancreatic adenocarcinoma: is cure possible? [J]. Ann Surg.

[8] Groot VP, Rezaee N, Wu W, Cameron JL, Fishman EK, Hruban RH (2018). Patterns, Timing, and Predictors of Recurrence Following Pancreatectomy for Pancreatic Ductal Adenocarcinoma [J]. Ann Surg.

[9] Kolbeinsson H, Hoppe A, Bayat A, Kogelschatz B, Mbanugo C, Chung M (2021). Recurrence patterns and postrecurrence survival after curative intent resection for pancreatic ductal adenocarcinoma [J]. Surgery.

[10] Shibata K, Matsumoto T, Yada K, Sasaki A, Ohta M, Kitano S (2005). Factors predicting recurrence after resection of pancreatic ductal carcinoma [J]. Pancreas.

[11] Zhang X, Feng S, Wang Q, Huang H, Chen R, Xie Q, et al. Comparative genomic analysis of head and body/tail of pancreatic ductal adenocarcinoma at early and late stages [J]. J Cell Mol Med. 2021;25(3):1750–8. 10.1111/jcmm.16281. 10.1111/jcmm.16281 PMC7875914 33452856

[12] Ling Q, Xu X, Zheng SS, Kalthoff H. The diversity between pancreatic head and body/tail cancers: clinical parameters and in vitro models [J]. Hepatobiliary & pancreatic diseases international : HBPD INT. 2013;12(5):480–7. 10.1016/s1499-3872(13)60076-4. 10.1016/s1499-3872(13)60076-4 24103277

[13] Sohn TA, Yeo CJ, Cameron JL, Koniaris L, Kaushal S, Abrams RA, et al. Resected adenocarcinoma of the pancreas-616 patients: results, outcomes, and prognostic indicators [J]. Journal of gastrointestinal surgery : official journal of the Society for Surgery of the Alimentary Tract. 2000;4(6):567–79. 10.1016/s1091-255x(00)80105-5. 10.1016/s1091-255x(00)80105-5 11307091

[14] Chun YS, Pawlik TM, Vauthey JN (2018). 8th Edition of the AJCC Cancer Staging Manual: Pancreas and Hepatobiliary Cancers [J]. Ann Surg Oncol.

[15] Zhang Y, Frampton AE, Kyriakides C, Bong JJ, Habib N, Ahmad R (2012). Loco-recurrence after resection for ductal adenocarcinoma of the pancreas: predictors and implications for adjuvant chemoradiotherapy [J]. J Cancer Res Clin Oncol.

[16] Habib JR, Kinny-Köster B, Bou-Samra P, Alsaad R, Sereni E, Javed AA (2023). Surgical Decision-Making in Pancreatic Ductal Adenocarcinoma: Modeling Prognosis Following Pancreatectomy in the Era of Induction and Neoadjuvant Chemotherapy [J]. Ann Surg.

[17] Maggino L, Malleo G, Crippa S, Belfiori G, Nobile S, Gasparini G (2023). CA19.9 Response and Tumor Size Predict Recurrence Following Post-neoadjuvant Pancreatectomy in Initially Resectable and Borderline Resectable Pancreatic Ductal Adenocarcinoma [J]. Annals of surgical oncology.

[18] Murata Y, Ogura T, Hayasaki A, Gyoten K, Ito T, Iizawa Y (2022). Predictive risk factors for early recurrence in patients with localized pancreatic ductal adenocarcinoma who underwent curative-intent resection after preoperative chemoradiotherapy [J]. PloS one.

[19] Seelen LWF, Floortje Van Oosten A, Brada LJH, Groot VP, Daamen LA, Walma MS (2022). Early Recurrence After Resection of Locally Advanced Pancreatic Cancer Following Induction Therapy: An International Multicenter Study [J]. Ann Surg.

[20] Sperti C, Pasquali C, Piccoli A, Pedrazzoli S (1997). Recurrence after resection for ductal adenocarcinoma of the pancreas [J]. World J Surg.

[21] Jones RP, Psarelli EE, Jackson R, Ghaneh P, Halloran CM, Palmer DH (2019). Patterns of Recurrence After Resection of Pancreatic Ductal Adenocarcinoma: A Secondary Analysis of the ESPAC-4 Randomized Adjuvant Chemotherapy Trial [J]. JAMA Surg.

[22] A G, Shan, Shan Y, Huo H, Ding C, Sun C. The Diagnostic Performance of 18F-FDG PET/CT in Recurrent Pancreatic Cancer: A Systematic Review and Meta-analysis [J]. Applied bionics and biomechanics, 2022, 2022:3655225. 10.1155/2022/3655225. 10.1155/2022/3655225 PMC9217608 35756868

[23] Morimoto D, Yamada S, Sonohara F, Takami H, Hayashi M, Kanda M (2020). Characteristics of Lung Metastasis as an Initial Recurrence Pattern After Curative Resection of Pancreatic Cancer [J]. Pancreas.

[24] Van Manen L, Groen J V, Putter H, Pichler M, Vahrmeijer A L, Bonsing B A, et al. Stage-Specific Value of Carbohydrate Antigen 19–9 and Carcinoembryonic Antigen Serum Levels on Survival and Recurrence in Pancreatic Cancer: A Single Center Study and Meta-Analysis [J]. Cancers, 2020, 12(10): 10.3390/cancers12102970. 10.3390/cancers12102970 PMC7602123 33066393

[25] Pausch T M, Liu X, Cui J, Wei J, Miao Y, Heger U, et al. Survival Benefit of Resection Surgery for Pancreatic Ductal Adenocarcinoma with Liver Metastases: A Propensity Score-Matched SEER Database Analysis [J]. Cancers, 2021, 14(1): 10.3390/cancers14010057. 10.3390/cancers14010057 PMC8750488 35008223

